# Breakfast consumption and physical activity in adolescents: daily associations and hourly patterns^1^,^2^,^3^

**DOI:** 10.3945/ajcn.111.027607

**Published:** 2013-11-27

**Authors:** Kirsten Corder, Esther MF van Sluijs, Charlotte L Ridgway, Rebekah M Steele, Celia J Prynne, Alison M Stephen, Diane J Bamber, Valerie J Dunn, Ian M Goodyer, Ulf Ekelund

**Affiliations:** 1 From the UK Clinical Research Collaboration Centre for Diet and Activity Research (KC and EMFvS), Medical Research Council (MRC) Epidemiology Unit (KC, CLR, RMS, EMFvS, and UE), University of Cambridge School of Clinical Medicine, Institute of Metabolic Science, Cambridge Biomedical Campus, Cambridge, United Kingdom; the MRC Human Nutrition Research, Elsie Widdowson Laboratory, Cambridge, United Kingdom (CJP and AMS); the Developmental Psychiatry Section, Department of Psychiatry, University of Cambridge, Cambridge, United Kingdom (DJB, VJD, and IMG); and the Department of Sports Medicine, Norwegian School of Sports Sciences, Oslo, Norway (UE).

## Abstract

**Background:** The association between breakfast consumption and physical activity (PA) is inconclusive.

**Objective:** We aimed to investigate daily associations and hourly patterns of PA and breakfast consumption in British adolescents.

**Design:** Daily PA [accelerometry-derived moderate and vigorous physical activity (MVPA)] and breakfast consumption (diet diary) were measured simultaneously over 4 d in 860 adolescents (boys: 43.4%; mean ± SD age: 14.5 ± 0.5 y). Associations between MVPA and breakfast consumption were assessed by using a multilevel mixed-effects logistic regression separately by sex and for weekends and weekdays. Hourly patterns of MVPA by breakfast consumption status were displayed graphically, and differences were tested by using ANOVA. Multilevel linear regression was used to investigate differences in log MVPA on days when 570 inconsistent breakfast consumers ate or skipped breakfast.

**Results:** On weekends, boys and girls with higher MVPA were more likely to eat breakfast [OR (95% CI): boys, 1.78 (1.30, 2.45) (*P* < 0.001); girls, 2.30 (1.66, 3.08) (*P* < 0.001)] when adjusted for socioeconomic status, percentage of body fat, and total energy intake. Peak hourly MVPA differed for breakfast consumers compared with nonconsumers on weekends (*P* < 0.001). Inconsistent breakfast consumers did more MVPA on days when they ate breakfast [exponentiated β coefficients (95% CIs): 1.2 (1.0, 1.5) on weekdays and 1.4 (1.1, 1.8) on weekends for boys and 1.6 (1.3, 2.1) on weekends for girls; all *P* < 0.03].

**Conclusions:** Eating breakfast was associated with higher MVPA on weekends. The time of peak MVPA differed between breakfast consumers and nonconsumers on weekends. Breakfast consumption at weekends is worth additional investigation to potentially inform PA promotion in adolescents.

## INTRODUCTION

Eating breakfast is widely considered to be important. Reviews that examined breakfast habits and body weight in youth concluded that skipping breakfast was associated with higher BMI (1, 2). Breakfast consumption has been positively associated with good academic performance, attention, and concentration (3, 4). In addition to benefits in youth, skipping breakfast may have implications for adult health (5). Although most evidence is cross-sectional (2), an Australian longitudinal study showed that participants who skipped breakfast in both childhood and adulthood had a larger waist circumference and higher fasting insulin, total cholesterol, and LDL cholesterol in adulthood than did those who ate breakfast at both time points (6).

Most studies in a systematic review showed that children and adolescents who are regular breakfast skippers were more likely to be overweight than regular breakfast eaters, despite a higher overall energy intake in the latter group (1). One explanation for the association between breakfast consumption and overweight is the clustering of breakfast skipping with other poor health behaviors, such as inadequate fruit and vegetable consumption (7). Breakfast skipping may also influence body weight through lethargy and reduced physical activity (PA)^4^ (3, 8, 9). Diet and PA are probably the 2 most important modifiable risk factors for overweight and obesity, but there has been relatively little research that has examined associations between these behaviors (10). Some studies have suggested that, compared with physically inactive individuals, physically active adolescents are less likely to skip breakfast and more likely to regularly eat breakfast (11–14). However, this relation is inconclusive, and several studies have shown no association (15–17). A diverse methodology may also partly explain varying results.

Many studies that assessed PA and breakfast consumption have used self-reported PA data and assessed habitual breakfast consumption by using a questionnaire, both methods of which may be prone to bias. The use of daily diet diaries over several days allows the matching of PA and breakfast-consumption data on a daily basis. To our knowledge, only one study in British 9–10-y-old children has examined PA and breakfast consumption on a daily basis, showing an association in boys but not girls (18). Our previous examination of habitual breakfast frequency in British adolescents indicated lower average PA over the morning in girls who reported being occasional breakfast consumers than in frequent consumers (10). To our knowledge, neither daily associations between matched PA and breakfast consumption nor hourly PA patterns in breakfast consumers and skippers have been investigated. Potential associations between PA and breakfast eating may differ by the time of day, being strongest in the morning, whereas other dietary behaviors may exert more influence later in the day. We aimed to investigate daily associations between PA and breakfast consumption and hourly patterns of PA for breakfast consumers and skippers in British adolescents.

## SUBJECTS AND METHODS

### Subjects

Volunteers were 860 adolescents (boys: *n* = 373; girls: *n* = 487; mean ± SD age: 14.5 ± 0.5 y), recruited to take part in the ROOTS study (19). ROOTS is a longitudinal cohort study established to determine the relative contributions that specific genetic, physiologic, psychological, and social variables make to overall risk of psychopathology during adolescence.

Secondary schools from Cambridgeshire and Suffolk, United Kingdom, in an area that extends 30 miles north, 20 miles south, and 20 miles west of Cambridge city, were approached. Of the 27 secondary schools invited, 18 schools agreed to participate (16 state and 2 independent schools). Study information, invitation letters, and parent and student consent forms were sent to eligible parents and students via the schools; 1238 adolescents who completed these forms at age 14 y were invited into the study. A second information sheet and consent form regarding the PA- and diet-assessment phase of the study were sent to adolescents attending at baseline; those adolescents who returned completed consent forms were invited to a measurement session at their schools. The current study used data from the first assessment of PA and diet, which was conducted ∼6 mo after baseline between November 2005 and July 2007. All procedures were verbally explained, and participants could choose to decline all or any part of the study. The full ROOTS study was approved by the Cambridge local research ethics committee.

### Measurements

Participants were visited at school by a team of trained researchers who administered the questionnaires, carried out the physical measurements, and gave instructions on dietary and PA measurements. Height was measured to the nearest 0.1cm (Leicester Height Meter; Invicta Plastics); weight was measured to the nearest 0.1 kg (Tanita TBF-300 MA; Tanita) in light clothing and without shoes and socks. Fat-free mass and fat mass were determined by using bioelectrical impedance (Tanita TBF-300 MA). Previously validated and published equations were used to derive the percentage of body fat (20).

### PA measurement

PA was assessed by using the Actiheart heart rate and movement sensor (CamNtech) (21–23). The Actiheart sensor clips onto 2 electrocardiography electrodes and was positioned in the midline just below the xiphisternum and attached via a 70–100-mm wire to a smaller clip horizontally to the left chest wall. Both parts were secured to the skin via standard electrocardiograph electrode pads. The Actiheart sensor recorded movement data in 30-s epochs. Participants were instructed to wear the monitor continuously for the remainder of the testing day and then for 4 consecutive days, including 2 weekend days. Participants returned the monitors to school for collection.

PA data were expressed as mean hourly and daily minutes of moderate and vigorous physical activity (MVPA). To find a MVPA cutoff comparable with that for the widely used Actigraph accelerometer (Actigraph LLC), a comparison between the Actiheart sensor and Actigraph 7164 accelerometer was done in a separate group of adolescents who wore both an Actigraph 7164 accelerometer and an Actiheart sensor while walking and running on a treadmill under controlled conditions (22). This laboratory study suggested a conversion factor of 5 (ie, Actigraph accelerometer counts = Actiheart sensor counts × 5), which was later confirmed in a free-living study of 254 12–17-y-olds (24).

After conversion, a custom program removed data recorded after 2300 and before 0600, periods of ≥60 min with continuous zero activity counts, and days with <600 min of recording (the cutoff for a valid day). The time (min/d) spent in MVPA was derived by using 2000 (Actigraph) counts/min as the lower threshold (25, 26). All minutes of MVPA were included. In regression models, MVPA was presented as hours per day for ease of interpretation. Participants were also classified as active or inactive by using a threshold of an average of 60 min MVPA/d according to British PA recommendations (5, 27). Mean hourly minutes of MVPA were used for graphical display of hourly patterns.

### Dietary assessment

Participants completed an estimated dietary record of all food and drinks consumed over a 4-d period during the school term, including 2 weekdays and 2 weekend days. Four-day diaries are the usual method used for surveys and large studies in the United Kingdom and were adapted from the previous 7-d diaries to incur a less respondent burden and, hence, increase the response rate. The 4-d diaries have been assessed alongside the gold-standard method of 7-d weighed records (28) and have been shown to produce comparable results. Four-day diaries are also the method used for the National Diet and Nutrition Survey in the United Kingdom, which is the Government funded national resource for nutrition information (29). Data were not corrected because they represent 4 complete days of intake. The diaries were divided into 7 time slots, the first of which was between 0600 and 0900 and designated as breakfast (30). The assessment period began on the day after the physical measurement session at school and was concurrent with the PA measurement. All participants received training on diet-diary completion during this measurement session when participants were asked to recall their last meal. Participants were asked to give estimated portion sizes in terms of small, medium, or large, household measures, or as individual items. Trained staff examined these recalls and instructed participants to add more information where necessary. Participants were asked to return the diet diary to school approximately 1 wk after the measurement. Participants needed ≥1 d of the matched diet and PA data to be included in the analysis. Returned completed diaries were coded by a group of experienced diet coders and editors, and the resulting intakes were analyzed for total daily energy and energy intake in the breakfast timeslot by using the Medical Research Council Human Nutrition Research in-house dietary assessment system Diet in Nutrients Out that is based on McCance and Widdowson's *The Composition of Foods*, sixth edition (31).

Breakfast consumption was dichotomized into 2 categories with skipping breakfast defined as an intake of 0 kcal between 0600 and 0900 (32), and subjects with an energy intake >0 kcal were classified as breakfast consumers for the main analysis. However, because of a lack of consensus on the best breakfast definition, we conducted sensitivity analyses by using a second breakfast definition that categorized participants with an energy intake ≥100 kcal between 0600 and 0900 as breakfast consumers. This definition was based on a literature review of definitions of breakfast from around the world (J Winter, M Chatfield, C Ni Mhurchu, R Hardy, A Stephen, unpublished observations, 2009) and was used to define subjects who consumed only 1 cup tea or coffee with milk and sugar (∼100 kcal) as nonconsumers of breakfast.

To establish comparability with other research that used a participant-defined definition of breakfast, we compared the diet-diary–derived breakfast consumption with a participant-defined habitual breakfast question. This question was at the front of the diet diary and was completed at the measurement session. Participants were asked “How many days a week do you usually eat breakfast?” Response categories were rarely or never as well as 1–2, 3–4, or ≥5 d/wk; the middle 2 categories were combined so that the variable had 3 categories of breakfast consumption frequency of never (0), sometimes (1), and always “2.” (10). For this comparison, diet diary data were also categorized as breakfast consumption on no days (0), some days [1 (some days of measurement], or every day “2” (all days of measurement).

### Socioeconomic status

A Classification of Residential Neighborhoods index was used as a proxy for socioeconomic status (SES) (33). A Classification of Residential Neighborhoods index is a postcode-based system that categorizes UK postcodes into 5 categories by using 125 demographic and 287 lifestyle variables (33). When SES data were unavailable, the category closest to the mean score for the school was used. Categories were combined to represent high (wealthy achievers and urban prosperity), middle (comfortably off), and low SES (moderate means; hard pressed).

### Statistical analyses

Differences in descriptive characteristics and PA by breakfast-consumption category (eating breakfast on no measurement days, some days, or all days) were assessed by using ANOVA, chi-square tests, or Kruskal-Wallis tests as appropriate.

Multilevel mixed-effects logistic regression that accounting for multiple measurements per participant and the nesting of participants within schools was used to examine associations between breakfast consumption (0 = nonconsumer; 1 = consumer) and PA. Models were adjusted for SES, percentage of body fat, and total daily energy intake. MVPA (h/d) was used as the independent variable for ease of interpreting results. Because we hypothesized that the MVPA × weekday/weekend interaction may differ by sex (10), we first tested a 3-way interaction between MVPA × weekday/weekend × sex. Because the *P* value for the 3-way interaction was = 0.02, we tested the 2-way interaction MVPA × weekday/weekend with the data stratified for boys and girls, and the interaction was significant for girls (*P* = 0.001) but not boys (*P* = 0.973). Therefore, we stratified analyses for both boys and girls and weekday/weekend for consistency. ORs reflected the change in odds of being a breakfast consumer per hour increase in MVPA. As a form of sensitivity analyses, these regressions were repeated by using the second breakfast definition of <100 kcal between 0600 and 0900 as breakfast skippers and ≥100 kcal as breakfast eaters.

To support or refute any associations identified from logistic regression models, data for the 570 inconsistent breakfast consumers were examined in more detail. An inconsistent breakfast consumer was defined as a participant with ≥1 d skipping breakfast and ≥1 d eating breakfast during the assessment period. The mean MVPA for the 570 inconsistent breakfast consumers was derived for days when breakfast was and was not eaten; this derivation was done for all days and separately for weekday and weekdays. Multilevel linear regression adjusted for school clustering, SES, percentage of body fat, and total energy intake were used to investigate differences between log MVPA on days when inconsistent consumers ate or skipped breakfast. Exponentiated β coefficients are presented for which any deviation from 1 indicated a percentage difference in MVPA for days when breakfast was eaten compared with days when breakfast was skipped.

**FIGURE 1.**
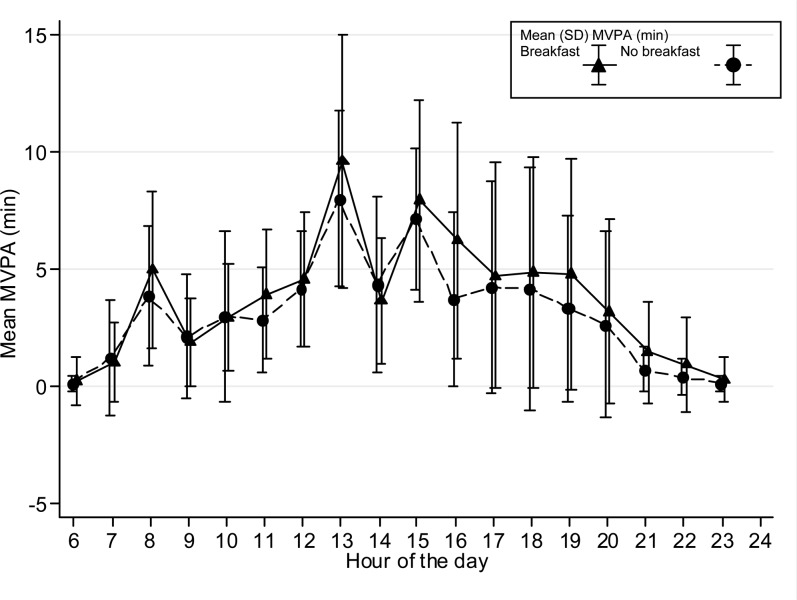
Hour-by-hour weekday mean (±SD) minutes of MVPA for boys on weekdays (*n* = 373). The time of peak MVPA did not differ significantly between breakfast consumers and nonconsumers. MVPA, moderate and vigorous physical activity.

**FIGURE 2.**
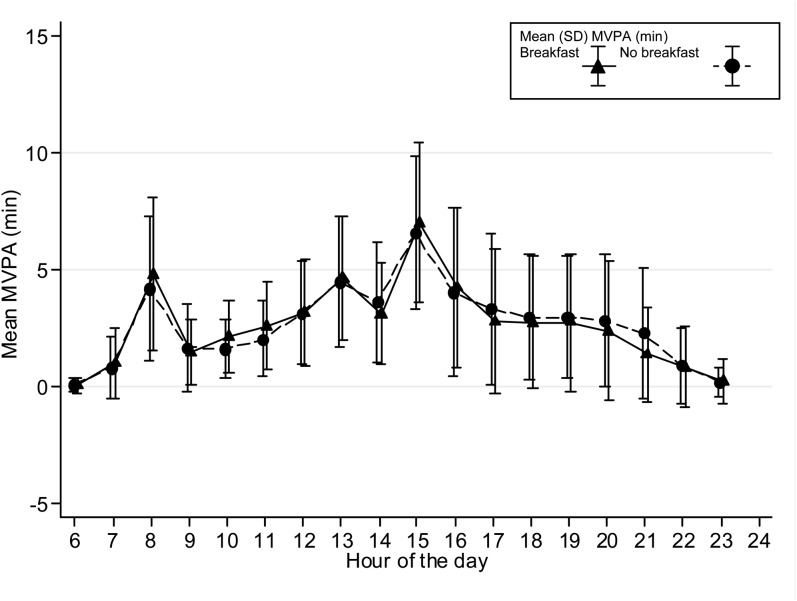
Hour-by-hour weekday mean (±SD) minutes of MVPA for girls on weekdays (*n* = 487). The time of peak MVPA did not differ significantly between breakfast consumers and nonconsumers. MVPA, moderate and vigorous physical activity.

**FIGURE 3.**
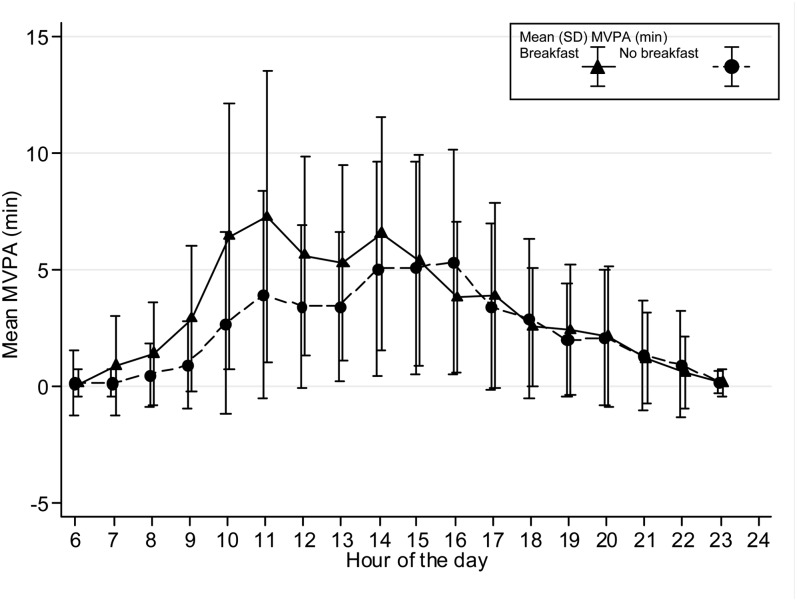
Hour-by-hour mean (±SD) minutes of MVPA for boys on weekend days (*n* = 373). The time of peak MVPA significantly differed between breakfast consumers and nonconsumers (*P* < 0.001). MVPA, moderate and vigorous physical activity.

**FIGURE 4.**
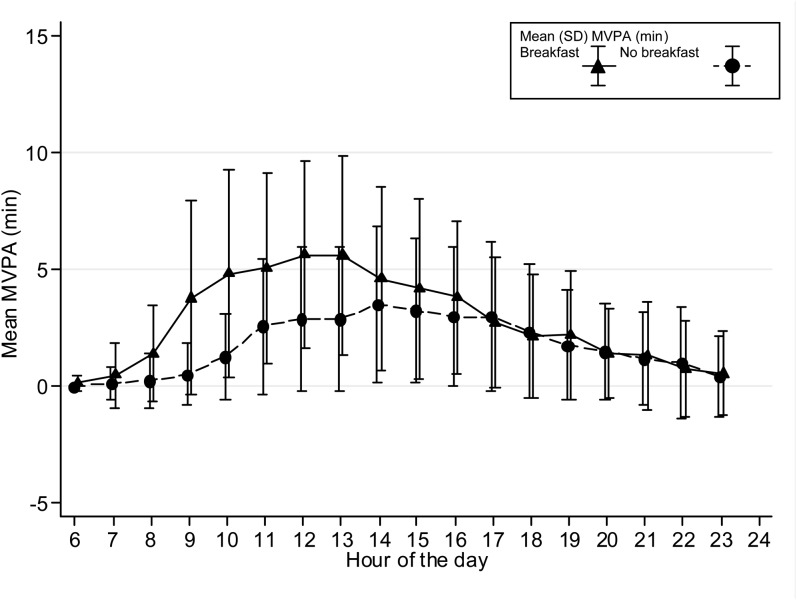
Hour-by-hour mean (±SD) minutes of MVPA for girls on weekend days (*n* = 487). The time of peak MVPA significantly differed between breakfast consumers and nonconsumers (*P* < 0.001). MVPA, moderate and vigorous physical activity.

The time spent in MVPA on an hourly basis is presented graphically stratified for breakfast consumers and nonconsumers and shown separately for boys and girls and weekdays/weekends. Only the hours between 0600 and 2400 are shown in **Figures 1–4** because of low night-time MVPA. To establish the difference in the timing of peak MVPA, ANOVA tests were conducted with the peak time for each subject used as the dependent variable and breakfast consumption as the independent variable. Analyses were stratified for boys and girls and weekdays/weekends.

The participant-defined and diet-diary–assessed breakfast-consumption variables were compared by using Cohen's kappa. Analyses were carried out with STATA 11 software (StataCorp).

## RESULTS

Of 1200 adolescents invited into the PA assessment part of the study, 998 adolescents (83%) and parents completed postal informed consent and 931 adolescents (93% of those consenting) attended a measurement session at school. There was no significant difference in SES (*P* = 0.60) or sex (*P* = 0.11) between subjects taking part in only the baseline assessment and subjects who were also measured in the PA and diet assessment. Compared with participants with complete data, subjects with missing PA or dietary data did not significantly differ by sex (*P* = 0.26), body weight (*P* = 0.86), body fat (*P* = 0.37), or SES (*P* = 0.97). Data from 860 adolescents (86.2% of those consenting and 92.4% of those attending a measurement session) with complete body-composition, PA, and diet-diary measurements were included in these analyses.

Descriptive characteristics are summarized in **Table 1**. Subjects who ate breakfast every day were more active when we examined minutes of MVPA (*P* = 0.005) but not when we examined adherence to PA recommendations (*P* = 0.545). On the basis of the 4-d food diary, boys were more likely to eat breakfast every day than girls were (*P* < 0.001). Height and percentage of body fat also differed by breakfast-consumption group with height positively and percentage of body fat negatively associated with breakfast consumption (*P* = 0.009 and *P* < 0.001, respectively).

**TABLE 1:** Characteristics of participants included in analyses stratified by the frequency of breakfast consumption*^1^*

	No days (*n* = 37)	Some days (*n* = 600)	Every day (*n* = 223)	*P*-difference
Age (y)	14.5 ± 0.5*^2^*	14.5 ± 0.5	14.5 ± 0.5	0.53
Sex (F) (%)	62.2	60.5	45.3	<0.001
Weight (kg)	56.7 ± 9.8	57.8 ± 11.0	56.9 ± 10.1	0.50
Height (cm)	166.3 ± 7.0	165.4 ± 10.7	167.8 ± 7.7	0.009
Percentage of body fat	19.1 ± 9.3	20.5 ± 9.5	16.7 ± 9.4	<0.001
Socioeconomic status (%)*^3^*				
Lowest	27.0	13.7	10.8	0.05
Middle	24.3	24.0	20.6	—
Highest	48.7	62.3	68.6	—
Physical activity				
MVPA (min)*^4^*	36.8 (27.8, 60.0)	42.8 (31.1, 62.3)	49.8 (33.8, 75.0)	0.005
No. of days of measurement meeting PA recommendations (%)				
No days	32.4	31.3	30.0	0.545
Some days	64.9	64.7	63.2	—
Every day	2.7	4.0	6.73	—

1 ANOVA, chi-square tests, or Kruskal-Wallis tests were used to determine differences for continuous and categorical variables, respectively. MVPA, moderate and vigorous physical activity; PA, physical activity.

2 Mean ± SD (all such values).

3 Derived from the home postcode (A Classification of Residential Neighborhoods category) scored from 1 (lowest socioeconomic status) to 3 (highest).

4 All values are medians; IQRs in parentheses.

Daily associations between breakfast consumption and MVPA are shown in **Table 2**. On weekdays, there were no significant associations between breakfast eating and MVPA. On weekends, breakfast consumption was positively associated with MVPA for both boys and girls when adjusted for SES, percentage of body fat, and total daily energy intake. Per 1-h increase in MVPA, odds of being a breakfast consumer increased at an OR (95% CI) of 1.8 [(.3, 2.5) (*P* < 0.001) for boys and 2.3 (1.7, 3.1) (*P* < 0.001) for girls. These analyses were replicated with breakfast consumption defined as the consumption of ≥100 kcal between 0600 and 0900, and associations were similar (data not shown).

**TABLE 2:** Associations between breakfast consumption and minutes of MVPA on a daily basis for boys and girls and for weekdays and weekend days separately*^1^*

	Boys	*P*	Girls	*P*
Weekdays				
MVPA	1.01 (0.99, 1.1)	0.311	0.97 (0.55, 1.69)	0.904
SES*^2^*				
Low*^3^*	—	—	—	—
Middle	2.47 (0.35, 17.5)	0.362	1.02 (0.35, 2.98)	0.973
High	3.86 (0.69, 21.8)	0.125	3.00 (1.10, 8.20)	0.032
Percentage of body fat	1.03 (0.93, 1.13)	0.610	0.97 (0.93, 1.02)	0.255
Total daily energy intake (MJ)	1.83 (1.40, 2.40)	<0.001	1.36 (1.20, 1.55)	<0.001
Weekends				
MVPA	1.78 (1.30, 2.45)	<0.001	2.30 (1.66, 3.08)	<0.001
SES*^2^*				
Low*^3^*	—	—	—	—
Middle	0.69 (0.27, 1.80)	0.454	1.31 (0.63, 2.71)	0.471
High	1.31 (0.57, 3.01)	0.518	1.37 (0.71, 2.63)	0.346
Percentage of body fat	0.99 (0.96, 1.04)	0.885	0.99 (0.67, 1.02)	0.789
Total daily energy intake (MJ)	1.14 (1.04, 1.24)	0.004	1.08 (1.00, 1.2)	0.047

1 All values are ORs; 95% CIs in parentheses. A multilevel mixed-effects logistic regression was used that accounted for the multiple measurements per participant and the nesting of participants within schools. The reference level was not eating breakfast. Models were adjusted for SES, percentage of body fat, and total daily energy intake (kJ). MVPA, moderate and vigorous physical activity; SES, socioeconomic status.

2 Derived from the home postcode (A Classification of Residential Neighborhoods category) scored from 1 (highest SES) to 5 (lowest).

3 Reference category.

Patterns of hourly MVPA for breakfast and nonbreakfast consumers are shown in Figures 1–4. The time of peak MVPA significantly differed between breakfast consumers and nonconsumers for both boys and girls on weekends (both *P* < 0.001) but not on weekdays.

Inconsistent breakfast consumers were defined as eating breakfast on some measurement days but not others (*n* = 570). On days that inconsistent consumers ate breakfast, boys did 20.5-min (95% CI: 12.0, 29.1-min) more MVPA, and girls did 13.7-min (95% CI: 8.4, 19.0-min) more MVPA (both *P* < 0.001) (data not shown). Results of the multilevel linear regression of the investigation of differences in MVPA on days when inconsistent consumers ate or skipped breakfast are shown in **Table 3**. The 25 boys who ate breakfast on some but not other weekdays were 24% more active on days when they ate breakfast; however, these differences were not significant for the 68 girls who ate breakfast on some but not other weekdays. The 237 participants who ate breakfast on one weekend day and not the other were 52% more active on the day when they ate breakfast (*P* < 0.001, respectively; ∼39% for boys and ∼63% for girls).

**TABLE 3:** Differences in MVPA on weekdays and weekend days when inconsistent consumers ate or skipped breakfast*^1^*

	Boys	*P*	Girls	*P*	All	*P*
Weekdays						
Inconsistent consumers (*n*)	25	—	68	—	93	—
Log MVPA (mean min/d)						
Skipped breakfast*^2^*	—	—	—	—	—	—
Ate breakfast	1.24 (1.03, 1.50)*^3^*	0.027	1.09 (0.90, 1.32)	0.353	1.15 (0.98, 1.34)	0.08
Weekends						
Inconsistent consumers (*n*)	97	—	140	—	237	—
Log MVPA (mean min/d)						
Skipped breakfast*^2^*	—	—	—	—	—	—
Ate breakfast	1.39 (1.08, 1.79)	0.015	1.63 (1.28, 2.08)	0.001	1.52 (1.28, 1.82)	<0.001

1 Multilevel linear regression was used and adjusted for the percentage of body fat, daily energy intake, socioeconomic status, and school clustering. Data are shown for the subsample who were inconsistent breakfast consumers (defined as ≥1 d skipping breakfast and ≥1 d eating breakfast during the assessment period). MVPA, moderate to vigorous physical activity.

2 Reference category.

3 Exponentiated β coefficient; 95% CI in parentheses (all such values). These values estimated the adjusted log scale MVPA mean difference between groups [ate breakfast compared with skipped breakfast (reference category)]; the antilog of exponentiated β coefficients was the ratio of geometric means [ate breakfast compared with skipped breakfast (reference category)]. Any deviation from 1 indicated a percentage difference in MVPA for days when breakfast was eaten compared with days when breakfast was skipped.

The comparison of participant-defined and diet-diary–assessed breakfast-consumption variables are presented in **Table 4** and showed an agreement of 41.6% (expected: 32.9%; Cohen's κ 0.13; *P* < 0.001), which classified as a slight agreement (34). The largest comparison group of participants (46.8%) stated that they always ate breakfast when asked the habitual participant-reported question but actually only ate breakfast on some measurement days when assessed by using the diet diary.

**TABLE 4:** Comparison data for 860 participants with both participant-defined habitual breakfast consumption and diet diary–assessed daily breakfast consumption

	Participant-reported habitual breakfast consumption			
	Never	Sometimes	Always
Diet diary–assessed daily breakfast consumption [*n* (%)]			
No days	22 (2.6)	17 (2.0)	0 (0)
Some days	55 (6.4)	141 (16.4)	403 (46.8)
Every day	5 (0.6)	20 (2.3)	197 (22.9)

## DISCUSSION

This study in 860 British adolescents that used simultaneous PA and breakfast assessment showed that breakfast consumption was associated with higher MVPA on weekends. The association between MVPA and breakfast consumption differed by the hour of the day on weekends.

The association between MVPA and breakfast consumption in boys and girls on weekends contrasts with results of previous studies that showed stronger associations between breakfast skipping and unhealthy behaviors in girls than boys (1, 9, 10, 12, 35, 36). This investigation of matched daily PA and breakfast consumption also somewhat contrasts with our previous results in this cohort, which showed no association between average PA and habitual breakfast consumption in boys, but less frequent breakfast consumption was associated with lower PA during the morning in girls (10). However, daily associations between PA and breakfast consumption in 9- and 10-y-old British children were only identified in boys (18).

To our knowledge, this study has investigated associations between breakfast consumption and PA in more depth than previously. There were nonsignificant associations between breakfast consumption and PA on weekdays for boys (*P* = 0.311) and girls (*P* = 0.904). Although these data did not support a sex difference in this association, we expected this association to differ by sex because girls report more dieting behaviors than boys and female dieters are 3 times more likely to skip breakfast than are nondieters (37). Girls are likely to both skip meals and increase their PA in pursuit of weight loss (38), which may complicate daily associations between these behaviors. Other factors, including different weight-control behaviors may also be associated with daily breakfast consumption in boys and girls. Compared with boys, girls have a higher prevalence of eating disorders (39), and girls with subclinical eating disorders are more likely to skip breakfast than healthy girls are (40).

Boys who were inconsistent breakfast consumers did 20.5-min more MVPA on days when they ate breakfast, whereas girls did 13.7-min more MVPA. This amount was ∼30% of total daily MVPA, and an increase in PA of this magnitude would be substantial. However, these differences were not shown on weekdays for girls. Only 63 girls ate breakfast on one weekday but not the other, and thus, a lack of power may have partly explained this lack of association. It is also possible that the mostly standardized requirements of a school day, such as seated lessons and walking between classes, may have meant that differences between the PA of breakfast-consumers and nonconsumers were less obvious during this time. At weekends, when adolescents could choose what to do, the association between MVPA and breakfast consumption was strongest. At weekends, adolescents may wake up later and, therefore, not eat between 0600 and 0900. However, hourly MVPA differences between breakfast consumers and nonconsumers on weekends appeared to remain until the early afternoon and, thus, were unlikely to have been solely a result of participants sleeping late. This finding may been more indicative of adolescents who had commitments, such as part time jobs or who played for sports clubs, and, hence, had greater levels of PA because they may have gotten up earlier and eaten breakfast in preparation. Unfortunately, data were not available to investigate what time adolescents got out of bed on a daily basis, which adolescents participated in a sports club, or which adolescents had a part-time job, but these data may have explained why some participants ate breakfast on weekends and were more active. Nonetheless, this analysis supports the hypothesis that skipping breakfast may lead to apathy and lethargy in the morning (3, 8, 9), but afterward, the association may weaken and possibly be influenced by later meals and nondietary factors. The results perhaps also suggest that health promotion that targets weekends could be especially effective. This effect may be of particular relevance for health because later sleep (rapid eye movement sleep) is negatively associated with obesity in adolescents and may be related to the suppression of feeding behavior (41).

On weekdays, there were no significant differences between breakfast groups in peak hourly MVPA, whereas on weekends, subjects who ate breakfast appeared to have higher peak MVPA than did nonconsumers. These differences may suggest that the association between breakfast consumption extends beyond the morning or that breakfast consumption clusters with other healthy behaviors. This result is in contrast with our previous findings in this cohort that showed no association between average PA and habitual breakfast consumption in boys (10). In the previous study, there was little heterogeneity in the habitual breakfast measure with only 5.8% of boys claiming to never eat breakfast (10). When assessed with a diet diary, 63.5% of these boys ate breakfast on some but not other days; this greater heterogeneity and increased power to detect an association may have partly accounted for differences seen with our previous results.

None of the associations observed in this study were attenuated by the addition of the percentage of body fat, SES, or total daily energy intake to models. This result adds support to the hypothesis that breakfast consumption may influence PA directly. Ideally, we would have investigated a variety of SES variables; however, there is evidence that the association between PA and diet is similar irrespective of the SES measure (42). Although our results appeared to be independent of total daily energy intake, other unstudied dietary behaviors, such as snacking, could be important. The increasing amount of literature examining objectively measured PA and well-measured dietary variables should help to clarify some of these uncertainties.

We classified breakfast as all food and drink reported to have been consumed between 0600 and 0900. It was possible that breakfast was eaten before or after this time, or that snacking behavior was included. The best way to define breakfast is equivocal; however, these associations were also shown when breakfast was defined as ≥100 kcal eaten before 0900. The diary structure was a limitation, and results may have differed if they were based on an alternative definition. However, this daily date-matched analysis and the examination of intraindividual variation in breakfast consumption were strengths that would not have been possible without a daily diary. Because breakfast consumption is often defined by the respondent and not researchers, it may be difficult to compare other breakfast studies with these results. Snacking between 0600 and 0900 could have contributed to the diet diary–defined breakfast consumption. Alternatively, the respondent-defined breakfast could have been prone to an ecological bias whereby behavior on the measurement day could have influenced reporting. Both of these factors were likely contributors to the observed slight agreement between participant-defined and diet diary–assessed breakfast consumption. When we compared these groups, 46.8% of the sample claimed to always eat breakfast but actually did not report food intake between 0600 and 0900 on some measurement days. Therefore, the observed slight agreement was likely not only the result of occasional snacking included in the diet-diary definition. In addition, the observation indicated the possibility that previous research that used participant-defined breakfast consumption has overestimated the frequency of breakfast consumption. Additional validation research is needed to establish the validity of breakfast measurement methods. An investigation of associations between different measures of breakfast consumption and physiologic or psychological health would be necessary to determine which metric is most strongly linked to health and, therefore, most worthwhile of future research.

Hourly MVPA patterns were similar to those in previous literature that examined hourly PA by the travel mode to school (43), which identified slightly higher MVPA before and after school. As suggested previously, this activity pattern could be characteristic of a clustering of healthy behaviors rather than a direct association between breakfast consumption and PA (12, 44, 45). This population had lower levels of overweight than did other adolescents from the East of England (46), and thus, results may not be generalizable to other British adolescents. Because this was a cross-sectional study, we could not imply causation from these results; only random assignment within a trial could determine whether skipping breakfast reduces PA. Although there were no differences between participants with complete data and those with missing data for anthropometric or demographic characteristics, it was possible that PA or dietary data may have differed and could have led to bias in our results. Compared with participants with complete data, subjects with missing PA or dietary data did not significantly differ by sex (*P* = 0.26), body weight (*P* = 0.86), body fat (*P* = 0.37), or SES (*P* = 0.97).

Our results suggested that breakfast could prove important in PA promotion. Additional work that investigates the association between PA and breakfast consumption and between PA and other dietary behaviors could be relevant for informing PA promotion interventions (47).

In conclusion, on weekends, eating breakfast is associated with higher MVPA. In addition, patterns of PA over the day on weekends appear to differ by breakfast consumption. Because of the limited evidence base, additional investigations of the association between PA and breakfast consumption could be useful. Breakfast consumption, especially at weekends, may be considered in adolescent PA promotion.

## Footnotes

4 Abbreviations used: MVPA, moderate and vigorous physical activity; PA physical activity; SES, socioeconomic status.
